# The effect of maxillary premolar distalization with different designed clear aligners: a 4D finite element study with staging simulation

**DOI:** 10.1186/s40510-024-00545-z

**Published:** 2024-12-02

**Authors:** Bochun Mao, Yajing Tian, Dawei Liu, Yanheng Zhou, Shuo Wang

**Affiliations:** 1 Department of Orthodontics, Peking University School and Hospital of Stomatology, Beijing, China; 2 China-Japan Friendship Hospital, Beijing, China

**Keywords:** Orthodontics, Clear aligner, Molar distalization, Finite element analysis

## Abstract

**Background:**

Molar distalization with clear aligners (CAs) is a common treatment. However, when the molars reach their target position and the distal movement of premolars begins, the mesial movement of molars might reduce the overall efficiency of molar distalization. This study aimed to investigate tooth movement patterns under different CA designs in the premolar distalization stage using a four-dimensional mechanical simulation method.

**Methods:**

A finite element method (FEM) model encompassing the maxillary dentition, periodontal ligaments, attachments, and associated CAs was constructed. The simulation aimed to replicate a premolar distalization of 2 mm within 10 sequential steps. Buccal interradicular mini-implants were used. Three groups of CAs were designed: the conventional CA design group (Con group), the second molar half-wrap group (SMHW group) and the all-molar half-wrap group (MHW group). An iterative computational approach was employed to simulate prolonged tooth movement resulting from orthodontic forces. Additionally, morphological alterations in the CA throughout the staging process were simulated utilizing the thermal expansion method.

**Results:**

Compared with the Con and SMHW groups, the MHW group presented significantly reduced mesial movement of the first and second molars. However, the MHW group presented the greatest displacement of canines and incisors. The distalization efficiency of premolars in the MHW group reached 95.5–96.5%, which was substantially greater than that in the Con group (84.5–85%) and the SMHW group (75–75.5%).

**Conclusions:**

The four-dimensional mechanical simulation results indicate that during the process of premolar distalization with CA, removing the distal portion of the aligner covering the first and second molars (MHW group) can effectively reduce the mesial movement of molars. Consequently, this approach can increase the overall efficiency of molar distalization.

**Supplementary Information:**

The online version contains supplementary material available at 10.1186/s40510-024-00545-z.

## Introduction

As a nonextraction orthodontic strategy, molar distalization can provide space for alignment or incisor retraction. Molar distalization with clear aligners (CAs) has been proven to be effective [1]. The accuracy of CAs for maxillary molar distalization has been reported to be as high as 87%^2^. However, a recent retrospective study revealed that the efficiency of molar distalization decreased to 42% following the subsequent anterior retraction process [3]. With respect to the biomechanics of molar distalization, several studies focused on the tooth movement pattern have proven that distalized maxillary molars achieve mostly tipping movement instead of the planned distal translation with CAs [4–6]. Molar distalization with different anchorage designs might have different effects on the whole dentition [7]. The use of temporary anchorage devices (TADs) combined with CAs can increase the efficiency of molar distalization [7, 8].

The classic whole treatment process of dentition distalization with clear aligners (DDCA), i.e., the ‘V-pattern’ strategy, can be divided into three stages [9]: (1) the molar distalization stage, in which molars are moved distally and space is moved anteriorly mesial to molars; (2) the premolar distalization stage, in which premolars are moved distally and space is moved anteriorly mesial to premolars; and (3) the aligning or retraction of anterior teeth, in which space in the anterior dentition is used to align or retract anterior teeth. During the staging process, the 3 stages are not exactly separate, with several steps in between that could belong to either stage.

In fact, molar distalization is only the first step of DDCA. In the subsequent treatment process, whether the space generated by molar distalization can be successfully transferred to the anterior arch region is a more important step in DDCA [10]. This determines whether the CA can achieve final success [11]. The efficiency of premolar distalization represents how much space has been transferred in this stage. However, there is currently a lack of research on the efficiency of premolar distalization and its underlying factors.

In recent years, finite element analysis (FEA) has been used in CA treatment simulations in various studies [4, 7, 12, 13]. Nevertheless, all prior studies conducted to date have focused solely on investigating the initial displacement observed during CA wear, thus overlooking the long-term alterations in dentition throughout CA treatment. The clinical significance of the results is further undermined by the fact that only one step of CA treatment was assessed in the FEA. Four-dimensional (4D) FEA, which considers the biomechanical response of the periodontal ligament (PDL) and the morphological changes in the CA during staging as another dimension, was introduced in our previous studies [4, 7], enabling long-term CA treatment simulation.

For DDCA, there is currently a lack of research on the premolar distalization stage. Therefore, this study used previously constructed 4D FEA of the DDCA-stimulating premolar distalization stage to observe the efficiency of the space forward transfer generated by molar distalization and to explore the impact of different aligner shapes on the efficiency of this stage.

## Methods

**Fig. 1:**
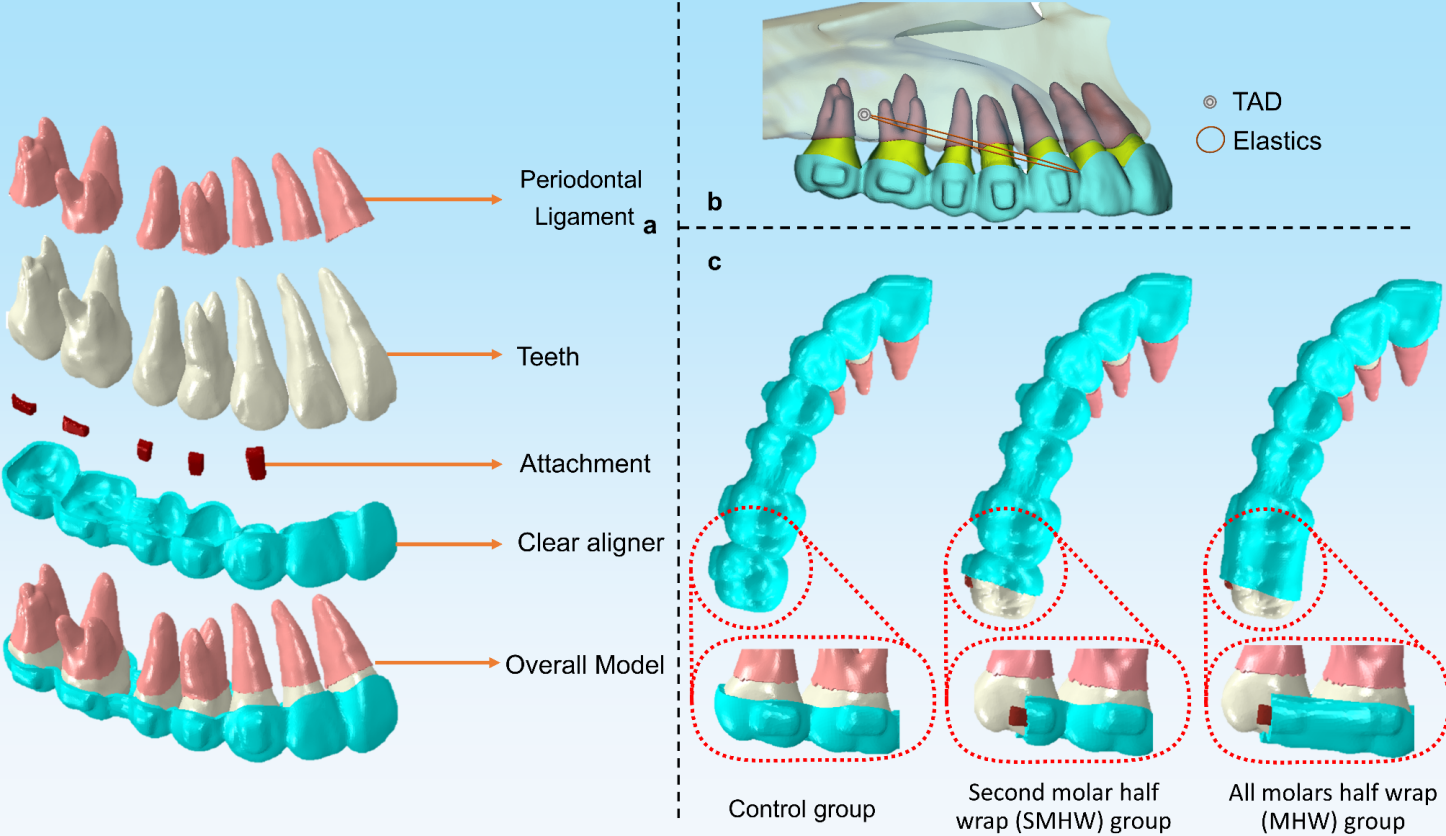
All parts of the model (**a**), the anchorage design (**b**) and the clear aligner design of different groups (**c**). TAD, temporary anchorage device

The Institutional Review Board of bioethics committee at the Peking University School and Hospital of Stomatology (No. PKUSSIRB-202059154) granted approval for this research. The FEA model contained teeth, PDLs, attachments, and CAs (Fig. 1a), generated from a patient’s cone beam CT (CBCT) and oral scan record using Mimics (Mimics 17.0, Materialise, Leuven, Belgium) and Geomagic Studio (Geomagic 15.0, 3D Systems, Rock Hill, SC), following the methodologies outlined in previous publications [4, 7, 12]. For computational efficiency, the model was constructed for the right side only, with symmetrical boundary conditions applied to the median section of the CA. A 0.30 mm shell element was utilized to represent the PDL [14]. Conventional rectangular attachments were designed.

A 2 mm space was set between the second premolar and the first molar (Fig. 1a), simulating the scenario where the two molars had completed distalization during DDCA. By employing the temperature changing method (TCM), ten steps of the distal body movement of the first and second premolars were designed, with 0.2 mm movement for each step. In other words, during the 10 steps, the CA between the first molar and the second premolar decreased by 2 mm, the CA between the first premolar and the canine increased by 2 mm, and both deformations were restricted in the mesial–distal direction.

The crowns were uniformly prepared to a thickness of 0.7 mm and preprocessed to create the initial CA model [15]. Elastic forces were applied to the CAs, extending from the buccal mesial cervical region of the canine to the buccal TAD, positioned within the buccal interradicular space between the first and second molars and situated 4 mm above the alveolar crest. The magnitude of the elastic forces was set at 150 g (Fig. 1b). In addition to the control group, which utilized a conventional CA design, two test groups were established, namely, (1) the second molar half wrap (SMHW) group, where the CA was cut off at the mid-coronal level of the second molar, and (2) the all molars half wrap (MHW) group, where the CA extended from the mid-coronal level of the first molar to the mid-coronal plane of the second molar, with the CA being cut off at the mid-coronal level of the second molar (Fig. 1c).

**Fig. 2:**
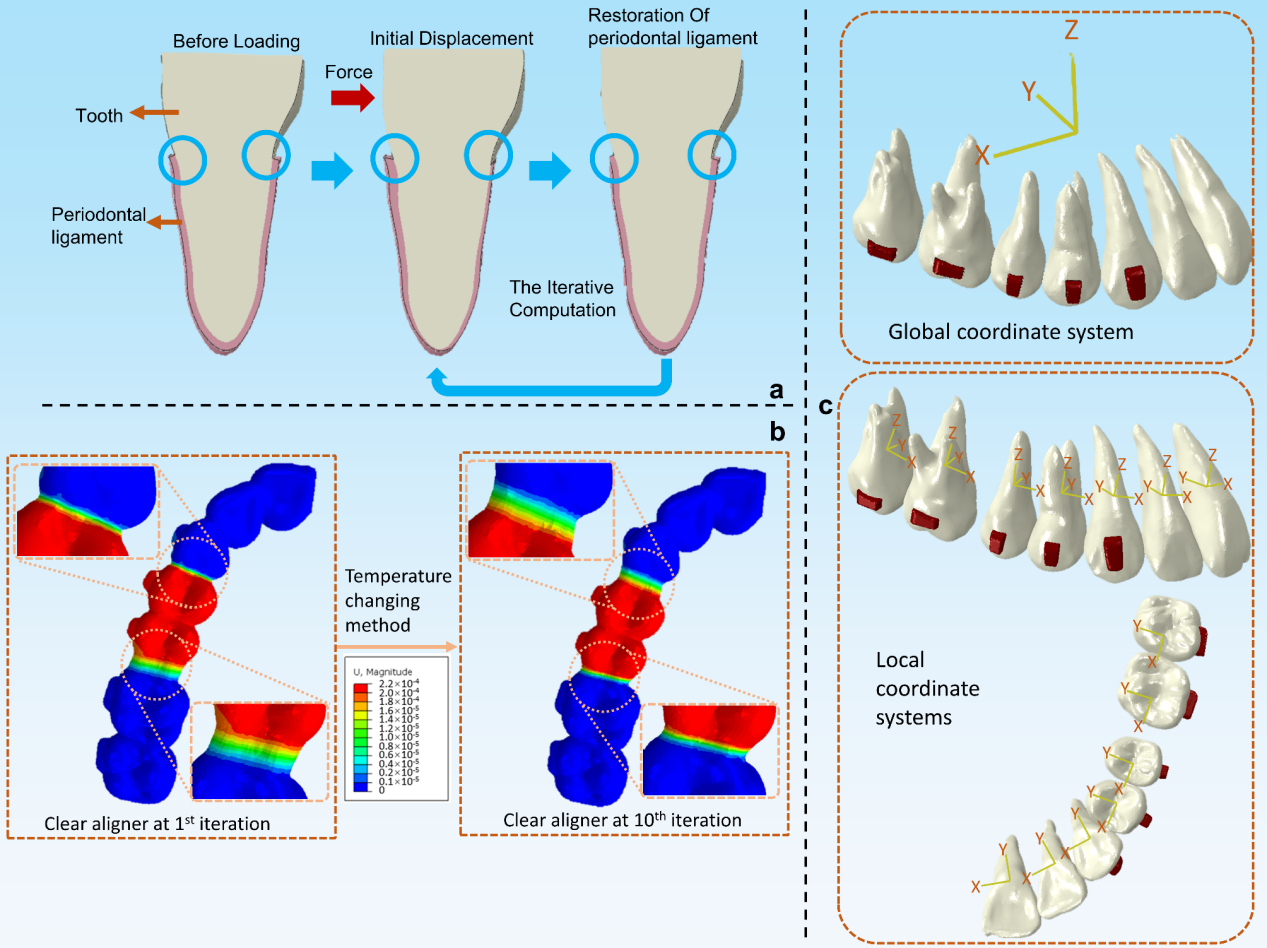
(**a**) Diagram of the algorithm simulating the bone remodeling process. The blue circles highlight the deformation of the periodontal ligament. (**b**) The temperature change method. (**c**) Two coordinate systems were used in this study to evaluate tooth displacement

As described in previous studies, an iteration method was employed to simulate the bone remodeling process during CA treatment (Fig. 2a) [4, 7]. In this iteration method, the alveolar bone and the teeth are simplified into rigid bodies without any deformation during the calculation. During the loading process, the PDL deformed. After the initial calculation, the displacements of the teeth were saved to the next calculation step, and the PDLs were deleted and regenerated for the next calculation step. Using the previously described TCM, automatic remodeling of the CA was implemented throughout the long-term orthodontic simulation (Fig. 2b, Supplementary Fig. 1, Supplementary Video 1) [4, 7]. A virtual CA was initially built for morphology changing to gain the solid model of ‘actual’ CAs for further 10 steps of simulation. Specific steps were detailed in Supplementary Fig. 1. During the TCM process, the CA between the canine and the first premolar increased by 0.2 mm in each step, and the CA between the second premolar and the first molar decreased by 0.2 mm in each step. The deformation direction and amount of CA were precisely controlled according to the formula detailed in Supplementary Fig. 1.

According to preliminary experimentation, less than 0.1% strain of the PDL was observed during the third iteration of the PDL adjustments at each stage of CA treatment, indicating that the model stabilized after the initial 2 iterations of the PDL [7]. Hence, to simulate the clinical scenario accurately, where CAs are worn over an adequate duration, each step of CA treatment was matched with two iterations of PDL adjustments during staging. Before each iteration, the CA from the previous step was removed and the CA for the next step was imported. Best-fit algorithm was carried out to match the inner surface of the CA with the dental crowns to simulate the wear-in process with custom Python subroutines.

The material properties for the involved models are detailed in Table 1 [16]. All of the models were assembled via Hypermesh 14.0 (Altair, Troy, Mich). Unstructured four-node tetrahedral elements were employed. ABAQUS/CAE (SIMULIA, Providence, RI) was subsequently used for FEA. The PDLs and tooth roots were considered position constraints. The contact relationship between the aligners and teeth was the same as that described in a previous study [17]. The optimal size of the elements was set as 0.2 mm according to the results of a convergence study.

**Table 1:** Parameters of the materials

Material	Elastic modulus (MPa)	Poisson’s ratio
Clear aligner	1,500	0.30
Attachment	20,000	0.30
Periodontal ligament	0.67	0.45

The occlusion plane and the global coordinate system were defined as described in a previous study [4, 7] (Fig. 2c). Local coordinate systems were used to illustrate the displacement of the teeth. The crown point (CP), root point (RP), resistance center (RC), and long axis (LA) of each tooth were determined, as shown in Table 2. For the local coordinate systems for each tooth, the Z-axes were aligned with the global coordinate system. However, the X- and Y-axes correspond to the mesial/distal and labial/lingual directions, respectively (Fig. 2d).

**Table 2:** Definition of the measurement indicators of each tooth during the analysis

Indicators	Definition
Crown point (CP)	The midpoint of the incisor edge, the cusp of the canine, and the midpoint of the occlusion surface of the premolars and molars.
Root point (RP)	The apex of the root of single-root teeth and the midpoint of root apexes of multirooted teeth
Resistance center (RC)	The midpoint of the section at the 1/3 root level for single-root teeth and the midpoint of the section at 1 mm above the furcation point for multirooted teeth.
Long axis (LA)	The line of crown point and root point for each tooth.

At the 10th step (target step), some remaining space was observed between the second premolar and the first molar. Therefore, a few more steps with the same prescribed teeth movements (0.2 mm distal movement per step for both premolars) were prescribed to completely close the remaining space, and the teeth movements at the ‘space-closed step’ of each group were also investigated.

To obtain more results with clinical significance, not only were the tooth movements at the 10th step compared but also a few more steps were prescribed to completely close the space between the second premolar and the first molar, and the tooth movements at the ‘space-closed step’ of each group were investigated.

## Results

**Fig. 3:**
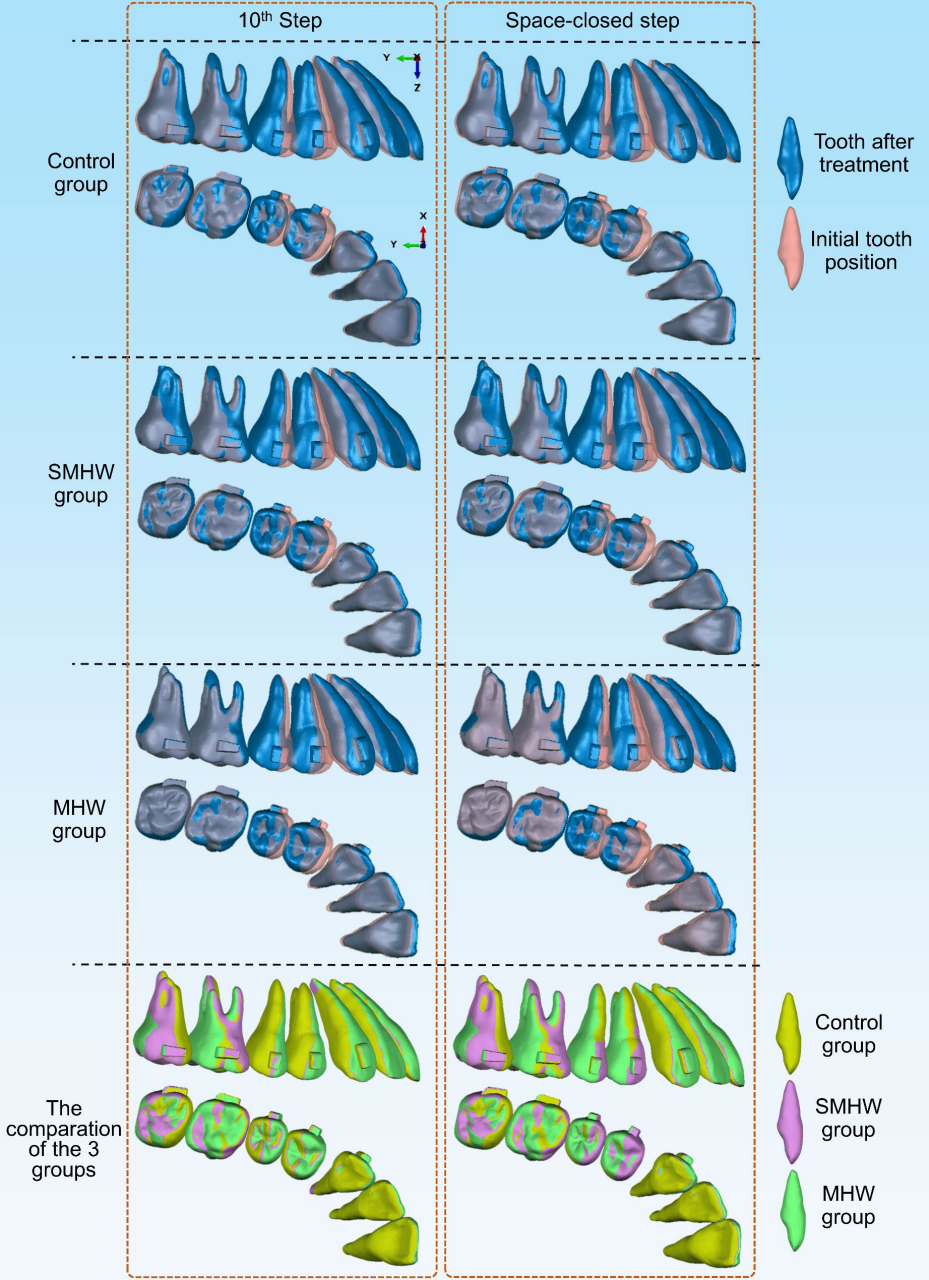
Tooth displacement of the 3 groups at step 10 and the space-closed step’. SMHW group, the second molar half-wrap group; MHW group, the all molars half-wrap group

**Fig. 4:**
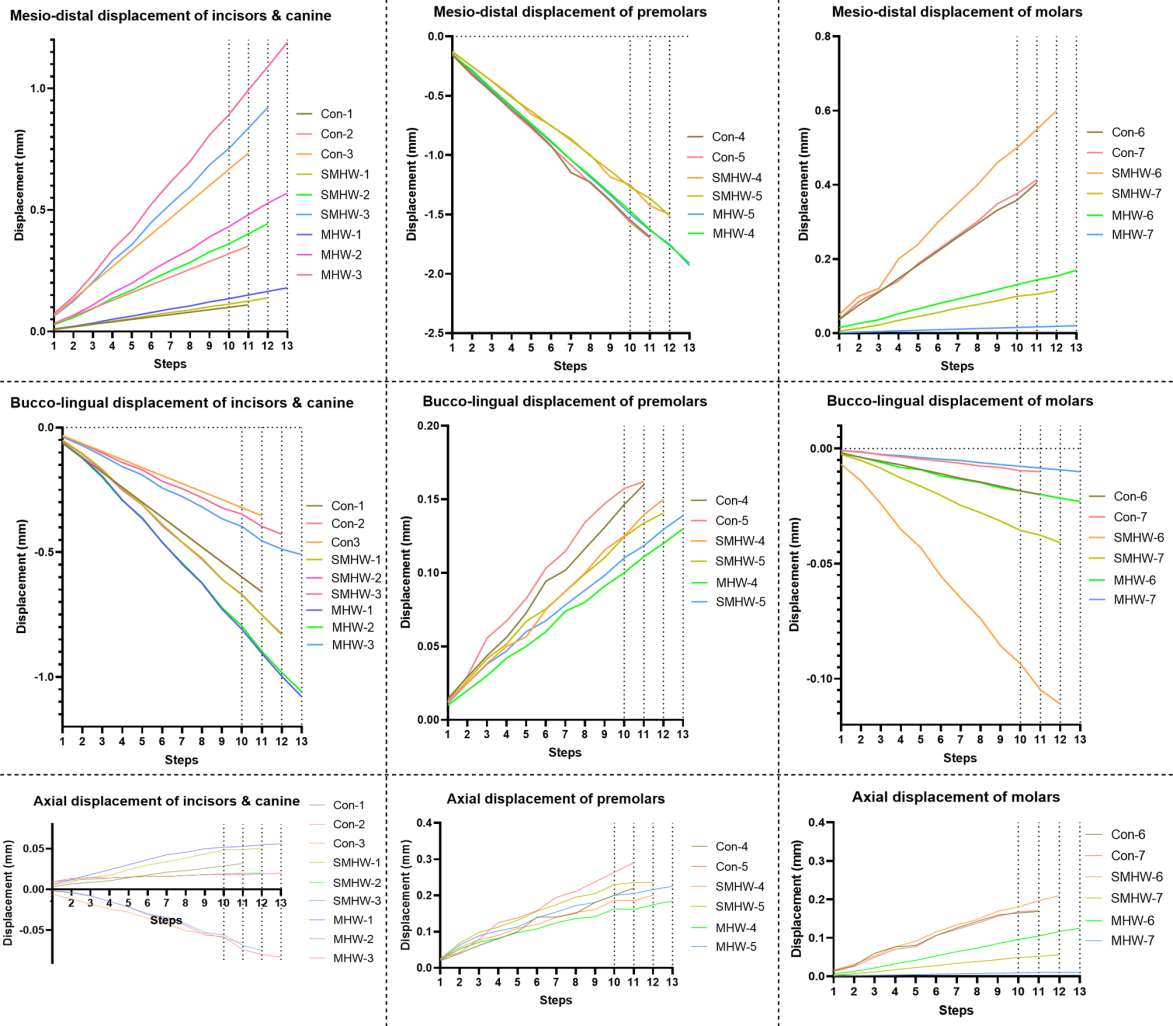
Within the local coordinate systems of each tooth, the crown displacements in the mesio-distal, bucco-lingual, and occlusal/gingival displacements of the teeth are shown. The tooth numbering follows the FDI tooth numbering system

**Fig. 5:**
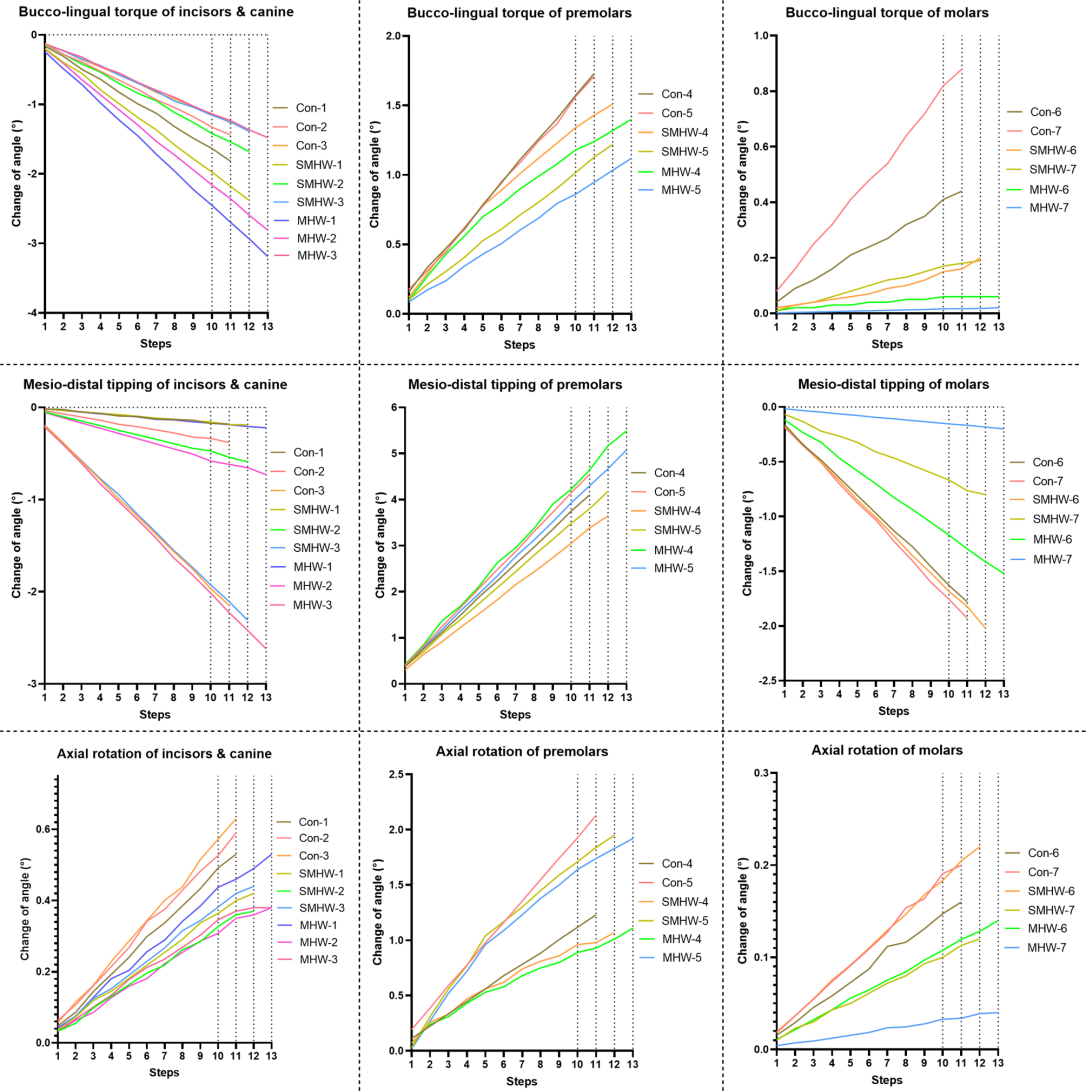
Within the local coordinate systems of each tooth, the buccal/lingual torque, axial rotation, and mesial/distal tipping of the teeth are also illustrated. The tooth numbering follows the FDI tooth numbering system

Supplementary Video 2 shows the tooth movements during each step in the 3 groups. The initial tooth position and the final position are shown in Fig. 3. The ‘space-closed step’ for the conventional CA design group, the SMHW group, and the MHW group was the 11th step, 12th step, and 13th step, respectively. Figures 4 and 5 illustrate the teeth displacement and rotation in local coordinates, respectively. In all line charts, steps 10, 11, 12, and 13 are marked with dashed lines to indicate the target movement step (10th step) and the ‘space-closed steps’ for all three groups.

All three groups experienced labial inclination and buccal movement of the canines and incisors in the ‘space-closed step,’ with the control group showing the least movement and the MHW group showing the greatest movement (Table 3). Specifically, the labial movement and inclination of the central incisors were 0.66 mm/1.82° (control group), 0.83 mm/2.38° (SMHW group), and 1.08 mm/3.19° (MHW group), respectively. For the canines, the mesial movement and mesial tipping were 0.73 mm/2.15° (control group), 0.92 mm/2.31° (SMHW group), and 1.19 mm/2.62° (MHW group), respectively.

**Table 3:** Three-dimensional displacements and rotation of teeth under the local coordinate systems at the ‘space-closed step’ of each group. The tooth numbering is according to the FDI tooth numbering system

Displacement (mm)/Rotation (°)		Groups	Teeth						
			1	2	3	4	5	6	7
Displacement of the crown point	Mesio-distal (X)	Con	0.11	0.35	0.73	-1.69	-1.70	0.41	0.41
		SMHW	0.14	0.44	0.92	-1.50	-1.51	0.60	0.11
		MHW	0.18	0.57	1.19	-1.91	-1.93	0.17	0.02
	Bucco-lingual (Y)	Con	-0.66	-0.66	-0.35	0.16	0.16	-0.02	-0.01
		SMHW	-0.83	-0.83	-0.43	0.15	0.14	-0.11	-0.04
		MHW	-1.08	-1.06	-0.51	0.13	0.14	-0.02	-0.01
	Axial (Z)	Con	0.03	0.02	-0.07	0.22	0.29	0.17	0.17
		SMHW	0.05	0.02	-0.07	0.20	0.24	0.21	0.06
		MHW	0.06	0.02	-0.08	0.19	0.23	0.13	0.01
Rotation of the long axis	Bucco-lingual torque (X)	Con	-1.82	-1.44	-1.27	1.73	1.71	0.44	0.88
		SMHW	-2.38	-1.68	-1.39	1.51	1.22	0.20	0.19
		MHW	-3.19	-2.81	-1.48	1.40	1.12	0.06	0.02
	Mesio-distal tipping (Y)	Con	-0.18	-0.38	-2.15	4.10	4.55	-1.78	-1.93
		SMHW	-0.19	-0.59	-2.31	3.65	4.18	-2.02	-0.80
		MHW	-0.22	-0.73	-2.62	5.49	5.08	-1.52	-0.20
	Axial rotation (Z)	Con	0.53	0.59	0.63	1.23	2.13	0.16	0.20
		SMHW	0.42	0.37	0.44	1.07	1.95	0.22	0.12
		MHW	0.53	0.38	0.38	1.11	1.92	0.14	0.04

Mesio-distal displacement: mesial (+) and distal (-); Bucco-lingual displacement: bucco (-) and lingual (+); Axial displacement: gingival (+) and occlusal (-); Buccal/lingual torque: buccal (-) and lingual (+); Mesial/distal tipping: mesial (-) and distal (+); Axial rotation: mesial rotation (+) and distal rotation (-); Con, control group; SMHW, second molar half wrap group; MHW, all molars half wrap group

In the ‘space-closed step,’ the MHW group exhibited the greatest distal tipping movement of the second premolars (1.93 mm/5.08°), followed by the control group (1.70 mm/4.55°), with the SMHW group showing the least (1.51 mm/4.18°). The SMHW group exhibited the greatest mesial tipping movement of the first molars (0.60 mm), followed by the control group (0.41 mm), with the MHW group showing the least (0.17 mm), and the trend at step 10 was the same. In the control group, the mesial tipping movement of the second molars was similar to that of the first molars (both 0.41 mm), with the second molars exhibiting mesial tipping of 1.93°. In contrast, the SMHW and MHW groups presented relatively less mesial tipping movement of the second molars (0.11 mm and 0.02 mm, respectively), with corresponding mesial tipping angles of 0.80° and 0.20°, respectively.

In this study, a molar distalization of 2 mm was designed. The initial space between the second molars and the first molars in all groups was set to 2 mm, resulting in an expected movement of 2 mm for the premolars. In each group’s ‘space-closed step,’ the efficiencies of the distal movement of the second premolars were 85.0% (control group), 75.5% (SMHW group), and 96.5% (MHW group). The first molars exhibited varying degrees of mesial tipping movement, resulting in a decrease in the achievement rate of the 2 mm movement, i.e., the loss of anchorage, during the distalization of the first molars, that is, 20.5% (control group), 30% (SMHW group), and 8.5% (MHW group). Thus, the design of the MHW group improved the overall efficiency of distalization of the molars by 12% during the step of distalizing the premolars by 2 mm.

**Fig. 6:**
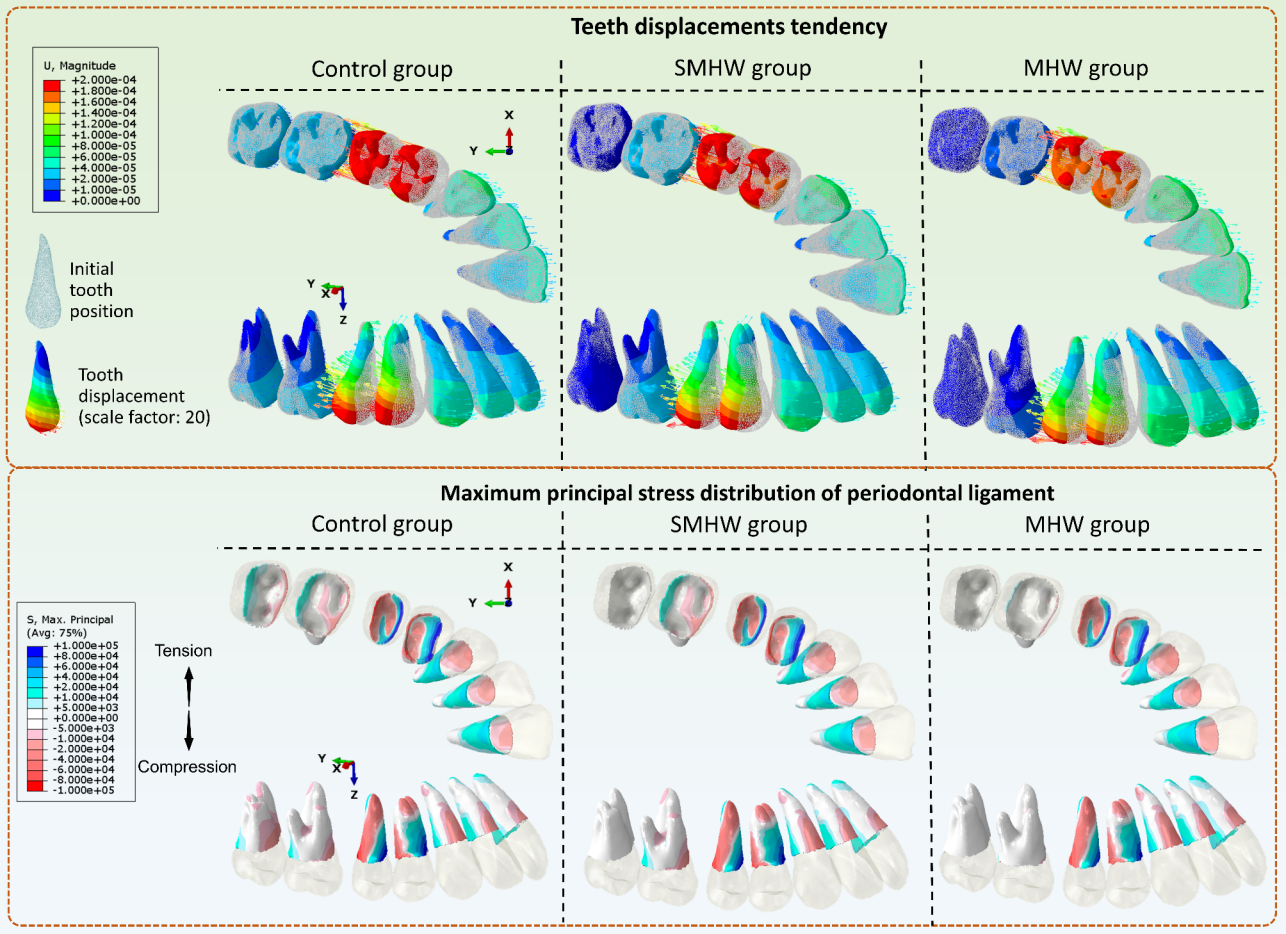
The displacement tendency of teeth with a scale factor of 20 and the maximum principal stress distribution of the periodontal ligament of all groups in the 1st step. SMHW group, the second molar half-wrap group; MHW group, the all molars half-wrap group

Using a scale factor of 20, Fig. 6 provides a more intuitive representation of the displacement tendencies of teeth, which is consistent with the numerical results in Figs. 4 and 5. Specifically, the second molars in the control group exhibited a noticeable tendency toward uncontrolled tipping, with the rotation center at approximately 1/3 of the tooth root apex. In the SMHW group, there was less unexpected movement in the second molars, whereas the first molars demonstrated greater mesial movement. Conversely, in the MHW group, there was an increased occurrence of labial tipping in the incisors and canines. The distribution of the maximum principal stress in the PDL (Fig. 6) further validates the aforementioned analyses, where cooler colors (blue) represent tension and warmer colors (red) represent compression.

## Discussion

Most FEA studies on CA have focused on a single step of CA treatment. However, the mechanical system in the CA treatment has a significant cumulative effect, where the complex interaction between the teeth and aligners determines the final result. The 4D FEA technique, which takes the biomechanical response as the 4th dimension, has been applied to orthodontic-related research in recent years [18, 19]. With 4D FEA, which enables the simulation of dynamically evolving interrelations among all components, orthodontists can obtain more substantial insights into long-term orthodontic treatment outcomes. Nevertheless, prior to our introduction of TCM to simulate the morphological changes in CAs [4], all 4D FEA investigations had been confined to fixed appliances. A pioneering simulation of long-term maxillary whole arch distalization with CAs was subsequently conducted for the first time in another study [7], in which the mesial movements of the pre-distalized molars were also noticed during the premolar distalization process.

There are two critical points of controversy surrounding DDCA: the movement pattern of the tooth and the overall efficiency. The movement pattern of molar distalization with CA represents varying degrees of tipping [5, 20]. CAs even showed better control of the vertical dimension and distal tipping of molars compared to fixed appliances [21]. To unify the baseline, the initial molar position was set as a total bodily distalization of 2 mm in this study, and premolars exhibited a similar movement pattern as molar distalization, with controlled bodily movement with varying degrees of mesiodistal tipping from 3.65° to 5.49°, increasing with moving distance. This result is consistent with the findings of a clinical study conducted by Yurdakul et al. [22]. Since tipping is challenging to avoid and ensuring sufficient distal movement of the premolars and molars is important in DDCA, it should be noted that there would be a greater relapse tendency for premolars and molars in cases where they tipped more.

Although molar distalization is considered one of the most predictable tooth movement patterns associated with CAs, the overall efficacy of DDCA has been debated among orthodontists [1–3, 7, 23]. Compared with the well-studied molar distalization stage, the biomechanical system of the premolar distalization stage has long been a neglected area of research. In the molar distalization stage of DDCA, a relatively simple force system is created where molars receive a distal pushing force while other teeth receive a mesial counterforce. This counterforce can be effectively countered by the TAD or intermaxillary traction [7]. Therefore, in research on the molar distalization stage of DDCA, regardless of the type of traction employed, high efficiency can be achieved [1, 7].

In this study, 2 mm distal movement was designed for the maxillary dentition. The amount of tooth movement was determined according to previous studies, which suggested that 2–3 mm distal movement of molars was a common target movement amount during DDCA [2, 3, 20, 24]. As molars reach their target position and the premolar distalization stage commences, the situation becomes more intricate than in the previous stage. For the premolars, the length of the CAs between the first molar and the second premolar is shortened, whereas the length between the first premolar and the canine is extended, resulting in a distal force. For the anterior teeth, a mesial counterforce is experienced. The most challenging issue involves molars. As the CA length between the first molar and the second premolar decreases, the molars experience a mesial force, and this force cannot be effectively countered. Consequently, this leads to mesial movement of the molars in subsequent stages, reducing the overall efficiency of DDCA.

**Fig. 7:**
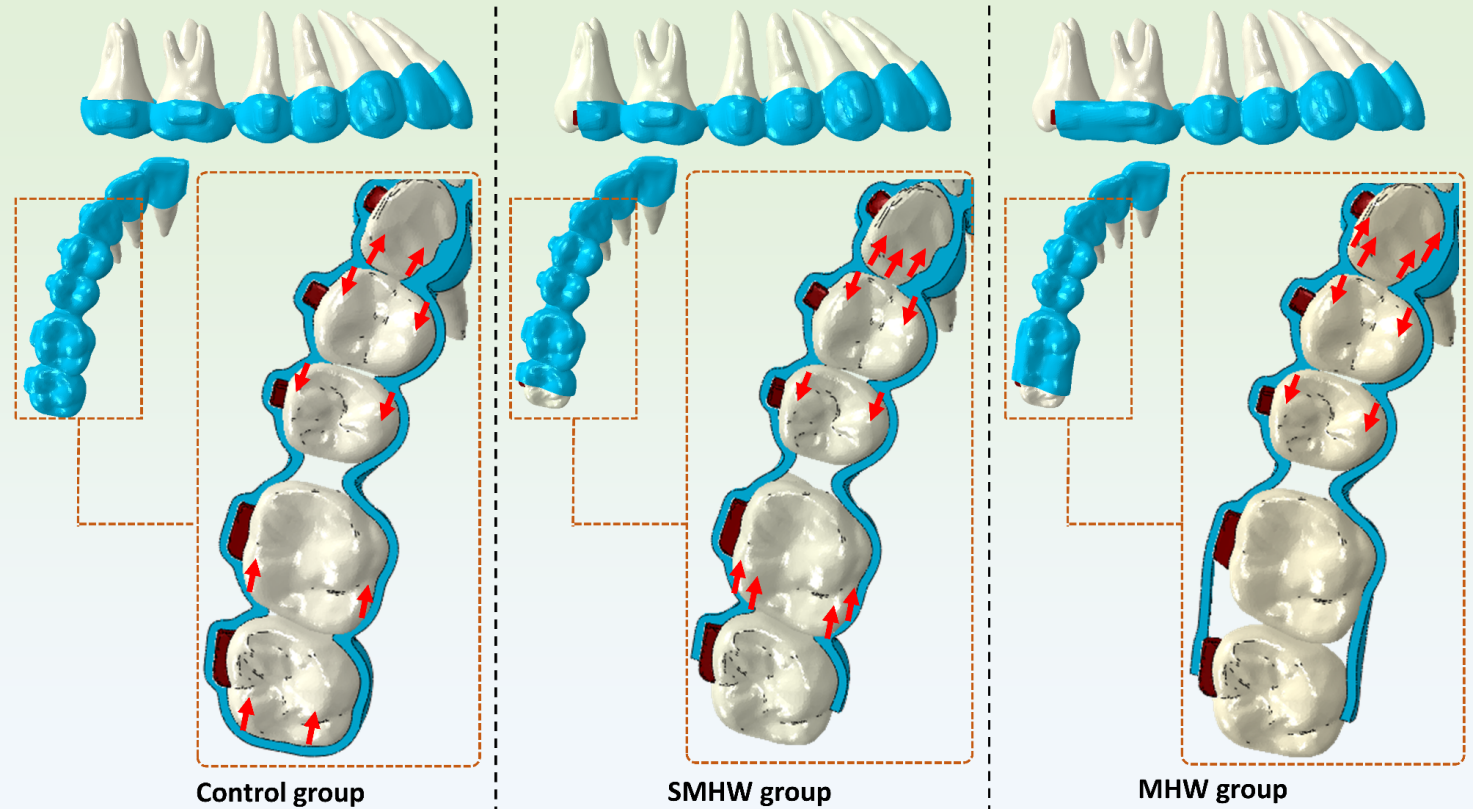
Schematic diagram of the mechanics of different designs of clear aligners. SMHW group, the second molar half-wrap group; MHW group, the all molars half-wrap group

This study compared the biomechanical effects of three different CA designs on the premolar distalization stages. Full-crown-surface-wrapped aligners (control group) are the current mainstream design for CAs. The first and second molars both experienced mesial force in the premolar distalization stage with this CA design (Fig. 7). In response to the undesirable counterforce on the molars, some manufacturers of CAs have removed the distal portion of the CAs for the second molar, which is the design of the SMHW group. The first molars still experienced mesial force on the premolar distalization stage with this design. In the MHW group, the CA design simultaneously removed the distal areas of the aligners that were in contact with both the first and second molars. This CA design eliminated the mesial counterforce produced by the ends of the aligners while retaining the ability of the CAs to block the mesial movement of the molars.

If contact between the second premolar and the first molar was considered the end of the simulation, the total number of steps for premolar movement varied among the three groups. This variation is due to the differences in the amount of mesial movement of the first molar among the three groups (Fig. 3). In the MHW group, the mesial movement of the first molar was the smallest, at only 0.17 mm, thus providing more distal movement space for the premolars and achieving a distal movement efficiency of 95.5–96.5%. This represents a significant increase compared with that of the control group (84.5–85%) and the SMHW group (75–75.5%). The unexpected inclination or rotation of the first and second molars was also lower in the MHW group (Fig. 5). Since the simulation assumed that at the start of the premolar distal movement, the first molars in all three groups were in the same 2 mm distally moved position, the baseline distal movement efficiency of the first molar was assumed to be 100%. The calculated molar distal movement efficiency at this stage, which is based on the final position of the first molar, represents the proportion of distal movement loss during this stage. The MHW group presented the lowest molar distal movement loss efficiency (8.5%), whereas the SMHW group presented the greatest loss efficiency (30%). However, it should be noted that in the MHW group, since neither the first nor the second molars provide anchorage in the mesial–distal direction, the movement of the incisors and canines was the greatest among the three groups (Fig. 3).

Notably, the mesial movement of the first molar was the greatest in the SMHW group (Table 3). This interesting phenomenon occurs because when the aligner of the distal part of the second molar is removed, the second molar no longer provides anchorage. Therefore, the force experienced by the first molar increases, leading to greater mesial movement of the first molar. The FEA model in this study did not incorporate calculations for relapse movement. However, clinical experience indicates that the second molar will relapse mesially with the mesial movement of the first molar. Additionally, compared with the full-crown-surface-wrapped CA design, the SMHW design increased the counterforce on the anterior teeth, resulting in increased mesial movement of the canines (Table 3). The original intention of this design was to reduce the mesial movement of the molars by eliminating the mesial counterforce by the end of the aligner on the second molar. However, as the results indicated, this design fails to prevent mesial movement of the molars and may contribute to an increased burden on the anterior teeth.

In this study, a buccal TAD, placed at the buccal interradicular space between the first and second molars situated 4 mm above the alveolar crest, was used for anchorage enhancement. Both the infrazygomatic crest and the interradicular space are suitable sites for buccal TAD placement. However, the buccal interradicular space is more commonly chosen due to its advantages in patient comfort and ease of clinical operation [8, 25]. With respect to the choice of the infrazygomatic crest, a larger amount of molar distalization could be achieved without worrying about the contact between the roots of molar and the TAD. However, larger buccal and vertical force components may lead to side effects [25]. With respect to the choice of the interradicular space, fewer buccal and vertical force components were gained, but a change in the location of the TAD may be needed with a greater degree of molar distalization [8]. In clinical practice, a feasible approach for buccal interradicular TAD placement during maxillary distalization involves positioning TADs between the premolars during the molar distalization phase. As the treatment progresses to distalize the premolars and anterior teeth, the TADs can be repositioned between the molars, as demonstrated in this study. As our previous study suggested, with a buccal TAD located in the interradicular space, during the DDCA process, the dentition is wider in the molar region, which may lead to unwanted side effects [7]. However, traction forces from TADs located in the infrazygomatic crest provide larger buccal and vertical force components, and the buccal component leads to palatal force in the molar region. Therefore, in the global coordinate system, for molars, the magnitude of the outward component of the distalization force and the inward component of the palatal force determine the horizontal movement tendency of the molar region. For this study, the inward component of the unwanted palatal force was relatively small due to the TAD location, which led to an increase in the width of the molar region. However, other situations need to be analyzed separately.

Conventional rectangular attachments were designed during the DDCA process in this model. However, novel attachment designs may further decrease the tipping tendency during distalization of the molars. Rossini et al. [26] carried out an FEA study of DDCA and concluded that attachments are mandatory during the distalization of the second molar. However, Hong et al. [27] suggested that different attachment designs had a limited impact on the efficacy of the designed movement during DDCA. Ayidaga et al. [28] analyzed the effects of different attachment designs on the efficacy of upper maxillary molar distalization, and their results indicated that vertical rectangular attachment significantly reduced the tipping tendency during distalization, whereas the novel attachment design they proposed offered the best control of molars. However, most of the current studies investigating different attachment types are limited to FEA, and further clinical evidence is needed.

Some shortcomings of this study must be noted. The first is the absence of calculations for the relapse tendencies of teeth, which is a critical issue that needs to be resolved in further FEA related to orthodontics. Moreover, several simplifications have been made in the current PDL iteration method to balance computational efficiency and accuracy [18], and errors might accumulate during the iteration process [29]. Only by combining further in vitro experiments and clinical trials could the accuracy of the 4D FEA could be certified and improved.

For the convenience of this study, DDCA was clearly divided into three distinct stages, which may differ from the specific clinical steps of DDCA [30, 31]. However, the findings of this study are significant, as they demonstrate that strategic removal of the distal part of the aligner for both the first and second molars can effectively curb the mesial movement of the molars in the premolar distalization stage. This, in turn, can enhance the distal movement of the premolars, a critical factor in improving the overall treatment efficiency of DDCA. The results of this study underscore the importance of simplifying the force applied to molars in this stage and focusing solely on restricting their mesial movement.

In future research, further consideration should be given to better controlling the positions of the incisors and canines to improve the treatment efficiency of DDCA further. Additionally, 4D FEA has shown great potential in CA treatment simulations. Since orthodontic treatment is a long-term process of complex tooth movement, dynamic simulation techniques that incorporate the effects of time can help us realize a broader range of clinical scenarios and contribute to clinical research in orthodontics.

## Conclusion

1. A 4D FEA model was developed to predict tooth movement during premolar distalization in the DDCA.
2. In the premolar distalization stage of DDCA, removing the distal portion of the aligner covering the first and second molars can effectively reduce the mesial movement of molars and increase the overall efficiency of molar distalization.
3. Elimination of the contour force on molars increases the proportion of anterior teeth in the premolar distalization stage.

## Electronic supplementary material

Below is the link to the electronic supplementary material.

Supplementary Figure 1: The temperature changing method. (**a**) The process is initiated by pinpointing the center point of the dental crown from the occlusal perspective, labeled (Ci, Cj). The margin points of the dental crowns along the Ci-Cj line were subsequently established as (Pi, Pj), after which the midpoint of Pi-Pj (Pc) was determined. An area perpendicular to the Ci–Cj line, encompassing both the mesial and distal regions to Pc, was selected as the deformation zone during staging. Deformation within this zone was constrained along the Ci–Cj line. In the formula, U represents the preset deformation magnitude, k represents the coefficient of linear expansion, Δ represents the deformation amount from previous steps, d represents the width of the area, and t represents the temperature change. (**b**) Process of gaining ‘actual’ clear aligners through temperature changing method. (**c**) The morphology of the virtual clear aligner and the ‘actual’ clear aligners of 3 typical steps

Supplementary Video 1: Occlusal and buccal views of the clear aligner over the course of 10 steps

Supplementary Video 2: Occlusal and buccal views of the dentition over the course of 10 steps

## Data Availability

No datasets were generated or analysed during the current study.

## Abbreviations

CA: Clear aligners
TAD: Temporary anchorage devices
DDCA: Dentition distalization with clear aligners
FEA: Finite element analysis
4D: Four-dimensional
TCM: Temperature changing method
SMHW: The second molar half wrap group
MHW: The all molars half wrap group
CP: Crown point
RP: Root point
RC: Resistance center
LA: Long axis
